# Babesiosis Presenting as Splenic Rupture in the Midwest: A Case Report

**DOI:** 10.7759/cureus.57659

**Published:** 2024-04-05

**Authors:** Heather L Mateja, Benjamin Yglesias, Esteban Tapias, Penelope Mashburn

**Affiliations:** 1 Surgery, American University of Antigua, Osbourn, ATG; 2 Surgery, Trumbull Regional Medical Center, Warren, USA

**Keywords:** syncope, emergent laparotomy, plasmapheresis, splenic rupture, babesiosis

## Abstract

*Babesia microti* is a parasite that invades erythrocytes inducing hemolysis. It presents with a variety of non-specific symptoms that can be mistaken for other illnesses. A rare manifestation of babesiosis is splenic rupture, generally seen in a younger, healthier population with low parasitemia, which can be treated conservatively depending on the grade and clinical condition. This case describes an elderly male with multiple comorbidities who is an avid hiker in the Northern Ohio and Western Pennsylvania areas presenting with a spontaneous American Association for the Surgery of Trauma (AAST) grade V splenic rupture requiring emergent splenectomy. Subsequent re-admission was required to diagnose babesiosis, which was managed with pharmacotherapy and plasmapheresis. In lieu of other identifiable etiologies, patients with atraumatic splenic rupture in an endemic area should be screened for possible parasitic infections.

## Introduction

*Babesia microti* is a protozoa transmitted primarily by the tick vector *Ixodes scapularis* but can occur through vertical transmission via transplacental, blood transfusion, or organ donation [1,2]. The initial trophozoite matures and reproduces inside erythrocytes leading to cell lysis, whereby free merozoites can invade other red blood cells [1]. Babesiosis is endemic in the Northeast and Upper Midwest United States, but there are many other species of Babesia that can be found globally [1].

Clinical manifestations range from subclinical to severe and may take weeks or months to manifest, as the incubation time following a tick bite is approximately one to four weeks [1,3,4]. Splenic rupture is a rare complication that usually presents in an otherwise healthy, younger patient with low parasitemia [2,3,5]. One study out of Rhode Island found that one in 100 patients with babesiosis will develop splenic rupture and are unlikely to require exchange transfusion after treatment [5].

It has been theorized that endothelial damage and release of local inflammatory factors may lead to necrosis of splenic tissue. This, in combination with enhanced hemolysis and excessive release of cytokines triggering red pulp hyperplasia, may all contribute to the stress and eventual rupture of the spleen [2]. Thus, healthier patients with a greater ability for phagocytosis within the spleen parenchyma may be at higher risk for splenic rupture, whereas those with low splenic function or asplenia may be at higher risk for high levels of parasitemia and more advanced disease, as they have an inability to clear the parasitic erythrocytes [2,4,5].

## Case presentation

A 70-year-old Caucasian male presented to the emergency department complaining of bilateral flank pain, sweating, nausea, and chills for three days. He reported having three syncopal episodes on the day of the presentation. Notably, during an orthostatic vital sign assessment, the patient had a witnessed syncopal episode necessitating assistance from staff. He denied any blunt trauma prior to the syncope, chest pain, shortness of breath, dysuria, or sick contacts.

His past medical history was significant for hypertension, hyperlipidemia, benign prostatic hyperplasia, asthma, gastroesophageal reflux disease, depression, anxiety, and schizoaffective disorder. He had just completed a five-day course of levofloxacin prescribed for suspected pneumonia. On abdominal examination, the patient had LUQ abdominal tenderness with distention, but the abdomen was soft without guarding or rebound tenderness. The cardiothoracic exam was unremarkable. Vital signs were borderline for hemodynamic stability with a triage blood pressure of 106/58 mmHg (recorded low of 99/22 mmHg), heart rate of 98 beats/min (recorded high of 112 beats/min), and respiratory rate of 18-22 breaths per min. He was afebrile with a room air oxygen saturation of 99%.

**Figure 1:**
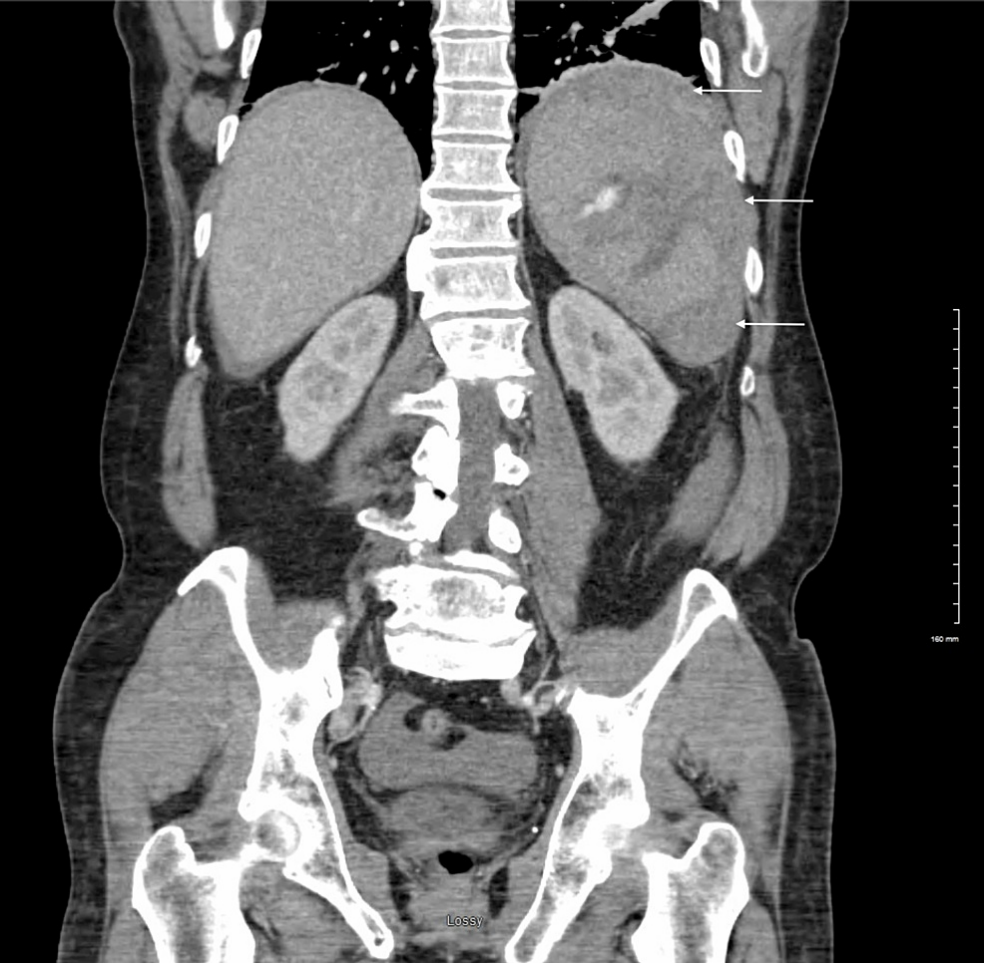
Coronal CT of the abdomen and pelvis with contrast showing a large area of hypodensity consistent with a splenic hematoma

**Figure 2:**
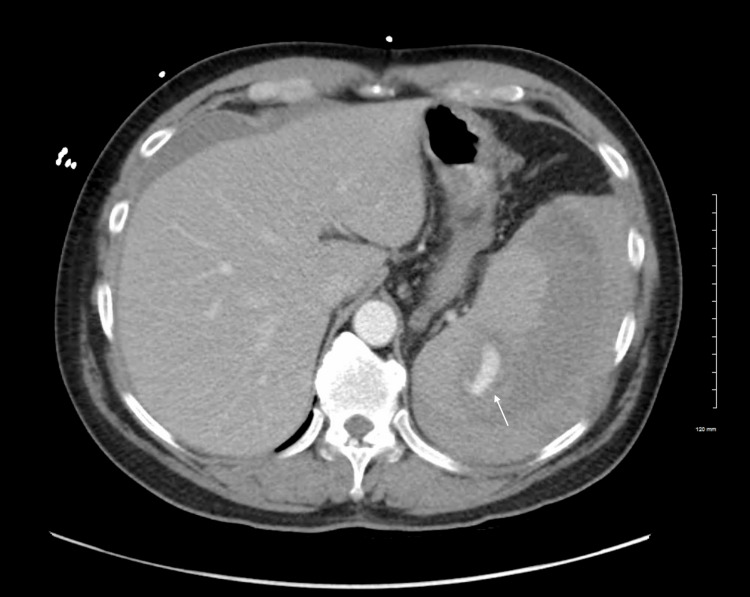
Axial CT of the abdomen and pelvis with contrast demonstrating a focal area of acute hemorrhage in the posterior aspect of the spleen measuring 2.8 × 1.4 cm

Initial laboratory data were significant for a hemoglobin of 9.8 g/dL, hematocrit of 28.7%, and platelet count of 63 × 10^3^/mL with a normal white blood cell (WBC) count of 9.3 × 10^3^/mL. Due to the recurrent falls with evidence of facial abrasions and loss of consciousness, a trauma protocol was initiated, and contrast-enhanced computed tomography (CT) scans were ordered for the face, head, thoracic spine, abdomen, and pelvis, during which it was noted the patient had a large splenic hematoma with active bleeding (Figures 1, 2).

The patient was taken for an emergent exploratory laparotomy and was found to have significant size enlargement of the spleen with a breach of the splenic capsule and multiple lacerations in the superior pole consistent with the American Association for the Surgery of Trauma (AAST) grade V classification. The spleen was removed via blunt dissection of all non-vascular ligaments, followed by transection of the hilum and short gastric arteries with the LigaSure and EndoGIA stapler. Suture ligation and Surgicel hemostatic powder were used to achieve hemostasis. The specimen was sent to pathology for further evaluation but did not return any remarkable findings associated with parasitic infection.

The patient was discharged on post-operative day (POD) 5 with post-splenectomy vaccines administered. During his follow-up office visit on POD 17, the patient reported having fevers (Tmax 102°F) with chills and a cough. He was placed on azithromycin without improvement and returned to the emergency department on POD 20. Laboratory values were significant for hemoglobin of 7.7 g/dL, hematocrit of 24.6%, an elevated WBC of 13.8 × 10^3^/mL with a neutrophil count of 7.73 × 10^3^/mL (18% bands) and a monocyte count of 1.52 × 10^3^/mL, procalcitonin level of 2.51 ng/mL, and lactic acid of 2.2 mmol/L. The pathologist notified the emergency department physicians that he had discovered a parasite in the blood and requested a blood smear to confirm. The smear confirmed *B. microti *infection, and the patient was started on 1000 mg azithromycin IV and atovaquone 750 mg orally twice daily.

Upon further questioning, the patient was an avid hiker in the Northern Ohio and Western Pennsylvania areas, and he had been on numerous trips weeks prior to his initial presentation where he had removed a few ticks. He denied feeling unwell prior to his presentation for syncope at the hospital. During his hospital course, the patient continued to have persistent hemolytic anemia, requiring multiple blood transfusions. He was found to have a parasitemia of 13%, necessitating transfer on hospital day two to another facility for plasmapheresis. After the exchange transfusion, the patient was able to be discharged from the hospital without recurrence of the anemia or detection of parasites.

## Discussion

Babesiosis is a growing concern across the United States as rates of transmission are climbing. This has been partly attributed to climate change, rising deer and tick populations, increasing proximity of humans and vectors, and better diagnostic methods [2,6]. It can be difficult to recognize babesiosis as the symptoms tend to be non-specific. Fever is one of the consistent features; however, malaise, fatigue, headache, cough, and arthralgias with or without hepatosplenomegaly may also be reported [5]. Laboratory abnormalities may also reflect hemolytic anemia, elevated lactate dehydrogenase, and, commonly, thrombocytopenia, as seen in our patient [5]. Unusually, our patient was older than the typical demographic for splenic rupture but did continue to have severe features with high parasitemia after splenectomy that required exchange transfusion despite proper pharmacotherapy.

In cases of atraumatic splenic rupture, causes may include neoplasm, infections, and inflammatory non-infectious conditions; however, *Babesia* is not one of the more likely pathogens [7]. Besides *Babesia*, *Plasmodium knowlesi* malaria infection may also need to be considered in malaria-endemic areas [8]. While the majority of atraumatic splenic ruptures are treated with splenectomy, this does not appear to be the same for Babesia-associated splenic rupture. In a 2018 review of atraumatic-pathologic splenic rupture secondary to *B. microti* infection, of the 12 reported cases, only three underwent splenectomy (25%), while the majority were managed conservatively [7]. Being that we did not initially suspect babesiosis and our patient was borderline hemodynamically unstable with CT imaging supporting a high-grade splenic rupture, splenectomy was the appropriate choice. 

The infection was unable to be identified until an experienced pathologist with expertise in parasitic infections noted the parasite in a blood sample prior to the blood smear after hospital re-admission. This raises concerns about the detection of uncommon causes for atraumatic splenic rupture prior to further progression of the disease, as they do not have typical manifestations of babesiosis. In such cases, diagnosis via splenectomy, when it is the first manifestation of the disease, is uncommon. A similar case report from 2018 describes the diagnosis of babesiosis in Wisconsin after re-admission following splenectomy [7]. While advances in diagnostic techniques have become widely available, a high clinical suspicion must be maintained for those in endemic areas, particularly when atraumatic splenic rupture does not have an identifiable cause. Some studies identify a mortality rate of 5% in patients with babesiosis, which may be higher in those who are elderly, immunocompromised, or have high parasitemia [2,9].

## Conclusions

Few case reports describe spontaneous splenic rupture occurring secondary to babesiosis in the Upper Midwest region, and it remains unknown to many clinicians as a possible differential. In this case report, we described an elderly male with multiple comorbidities who first presented to the hospital with an AAST grade V splenic rupture requiring emergency splenectomy and was later found to have babesiosis with a parasitemia of 13% requiring plasmapheresis after failed pharmacotherapy management. Patients with atraumatic splenic rupture may benefit from parasite screening with a blood smear to prevent further progression and other complications associated with increasing levels of parasitemia.
